# Behçet’s disease and avascular necrosis of the knees: a case report

**DOI:** 10.1097/MS9.0000000000004554

**Published:** 2025-12-18

**Authors:** Meera Abou Dehn, Wael Barakeh, Sati Dib, Oussama Nasrallah, Riad Khouazami, Mohamad Ali Rida, Mira Merashli

**Affiliations:** a Faculty of Medicine, American University of Beirut, Beirut, Lebanon; b American University of Beirut Medical Center, Beirut, Lebanon; c Department of Rheumatology, Clemenceau Medical Center, Beirut, Lebanon

**Keywords:** avascular necrosis, Behçet’s disease, case report

## Abstract

**Introduction and importance:**

Behçet’s disease (BD) is a rare, autoimmune vasculitis usually characterized by oral and genital ulcers with systemic involvement of multiple organ systems. Its diagnosis is usually clinical as it lacks the disease-specific markers seen in other autoimmune pathologies. Avascular necrosis (AVN) is extremely rare in BD and if it were to occur, it would usually be unifocal.

**Case presentation:**

This case report presents an odd case of BD that was complicated by bilateral AVN of the knees in a 36-year-old man. His diagnosis was confirmed by MRI of both knees after he started complaining of knee pain while on rituximab. The patient’s treatment was adjusted and he has reported relief from pain and clinical improvement while on Infliximab, which he is currently maintained on.

**Clinical discussion:**

AVN is usually observed with chronic steroid use or with diseases such as sickle cell disease. The vasculitis associated with BD may result in an ischemic insult to the bone and joints; however, the pathophysiology is still unclear. Hence, joint pain in BD patients may be a warning sign to AVN which thus needs to be considered among the differential diagnosis in these immunosuppressed patients.

**Conclusion:**

This is case of bilateral AVN of the knees in a patient with BD warrants investigation of joint pain or symptoms in patients with BD and other autoimmune diseases even if not on corticosteroid treatment. Prompt evaluation of patients’ symptoms decreases risks of development of serious complications and preserves their health.

## Introduction

Behçet’s disease (BD) is a chronic, inflammatory, autoimmune vasculitis of unknown etiology, primarily affecting individuals of Middle Eastern and Far Eastern descent. It manifests predominantly with recurrent oral and genital ulcers, alongside systemic involvement of multiple organ systems^[1]^. First described by Turkish dermatologist Hulusi Behçet in 1937, BD most commonly affects young adults aged 20 to 40, with a male predominance observed in Arab populations^[2,3]^. The disease tends to follow a more severe course in males and younger individuals^[3]^.

The high prevalence of BD in Middle and Far East suggests a strong genetic component, with the HLA-B51/B5 allele being the strongest known risk factor^[4]^. However, BD does not follow a Mendelian inheritance pattern and is believed to be triggered by environmental factors and infections in genetically predisposed individuals^[5,6]^. Pathologically, BD is characterized by a neutrophilic vasculitis affecting arteries and veins of all sizes, leading to endothelial swelling and fibrinoid necrosis, but lacking the necrotizing vasculitis or giant cell formation seen in other vasculitides^[1]^. Despite its autoimmune nature, BD does not present with disease-specific autoantibodies, making its diagnosis primarily clinical^[7]^.

**HIGHLIGHTS**

- 36-year-old man with Behçet’s presents with bilateral knees’ avascular necrosis.
- Patient was on treatment with rituximab.
- Early recognition is critical to avoid joint damage and loss.
- Control of disease severity is essential to ensure good quality of life.

While mucocutaneous manifestations are the hallmark of BD, the disease can lead to severe systemic complications, including uveitis, large-vessel vasculitis, and neurological involvement with the latter presenting as neuro-Behçet a rare, yet debilitating disease^[8,9]^.

Avascular necrosis (AVN) or osteonecrosis is a common occurrence in autoimmune and rheumatological diseases often as a complication of chronic corticosteroid use^[10]^. Unlike other rheumatological diseases, AVN rarely occurs with BD. The pathogenesis of AVN in BD is hypothesized to be linked to its prothrombotic state, leading to vascular occlusion and subsequent ischemia^[11]^. Although rare, AVN cases with BD have been reported in patients on corticosteroids. The cause of AVN was not clearly determined to be related to either corticosteroid use or the vasculitis and thrombosis of BD^[10]^. We present a unique case of a 36-year-old man known to have BD, who presented with bilateral osteonecrosis of the knees in the absence of corticosteroid use. This article would significantly contribute to the limited literature on this unusual association, as the available literature discusses BD and AVN in patients who have been on high-dose corticosteroid unlike our patient who developed his symptoms when he was on rituximab^[12]^. Furthermore, the available literature describes BD AVN that involved one knee joint only unlike bilateral knees in our patient^[13]^. This case report has been reported in line with the SCARE checklist^[14]^.

## Case

This is a case of a 36-year-old man who initially presented 22 years ago with recurrent aphthous ulcers and one episode of genital ulcers and was diagnosed with BD. The patient was started on cyclophosphamide, and 2 months prior to presentation, treatment was switched to rituximab.

Regarding his treatment history, the patient previously received a course of cyclophosphamide (500 mg every month for three doses), which was later switched to rituximab (1 g for two doses, given 2 weeks apart). During this period, he was maintained on colchicine (1 mg daily), azathioprine (150 mg per day), acenocoumarol, and nifedipine (30 mg daily).

Six years prior to his current presentation, the patient presented with a new-onset discoloration of the toes on his left foot, accompanied by burning pain. The patient was on colchicine and azathioprine when he developed severe toe pain showing osteomyelitis visualized on imaging. This was preceded by painful and numerous oral aphthous ulcers without SICCA symptoms (xerophthalmia and xerostomia) or genital ulcers. The patient was by then due for his cyclophosphamide dose which he received and improved on. However, the patient developed skin and soft tissue infection (SSTI) 2 months later that was managed with doxycycline that resolved the SSTI. The patient was cleared to resume cyclophosphamide again; however, his toe pain recurred which prompted the decision to switch treatment to rituximab. A few months after rituximab initiation, the patient reports fatigue and right knee pain that had later progressed to both knees. No swelling or trauma was reported. The pain was not relieved by rest or movement. This pain was not reported by the patient when he was treated with cyclophosphamide in addition to the improvement of his toe pain on the latter treatment.

Laboratory studies were performed to rule out other autoimmune diseases. cANCA, pANCA, anti-SSA, anti-SSB, antinuclear antibodies (ANA), and anti-cyclic citrullinated peptide (anti-CCP) were all negative as shown in Table 1. Additionally, the erythrocyte sedimentation rate (ESR) was 10 mm/hour, and C-reactive protein (CRP) was 17.6 mg/L.

**Table 1:** Summary of patient’s serology workup during hospital admission

Test	Patient Values	Reference range
ANA	Negative	Negative
Anti SSA (U/mL)	<15	Normal <15
Anti SSB	<15	Normal <15
pANCA (U/mL)	<5 U/ml	Normal <5 U/ml
cANCA (U/mL)	<5 U/ml	Normal <5 U/ml
Anti cardiolipin IgM	<7 mPU/ml	Normal <7 mPU/ml
Anticardiolipin IgG	<10 GPU/ml	Normal <10 GPU/ml
Lupus anticoagulant	Negative	Negative
Anti B2 glycoprotein IgM	<5 U/ml	Normal <5 U/ml
Anti B2 glycoprotein IgG	<5 U/ml	Normal <5 U/ml
anti HCV antibodies	Negative	Negative
HbsAg	Negative	Negative
HLA B51, HLA B27	Negative	Negative
Cryoglobulin qualitative	Negative	Negative

**Figure 1.**
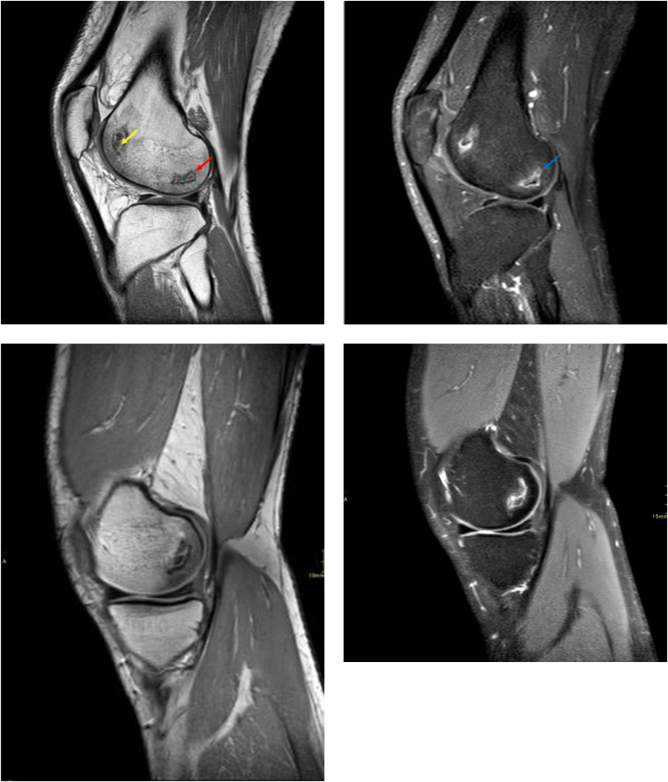
Sagittal T2-weighted image of the left knee shows geographic areas in the bone marrow of the lateral condyle of the distal femur, demonstrating the “double-line” sign, consisting of an outer sclerotic hypointense rim (red arrow) and inner hyperintense granulation tissue (yellow arrow), which is a pathognomonic MRI pattern seen at the periphery of an area of osteonecrosis, representing the boundary between viable and non-viable bone. Sagittal post-gadolinium fat-saturated T1-weighted image of the left knee shows contrast enhancement along the boundaries of the infarcted bone regions at the junction with viable bone tissue (blue arrow), and complete absence of contrast enhancement in the central necrotic area. Sagittal T2-weighted image of the right knee shows a geographic area in the bone marrow of the medial condyle of the distal femur, demonstrating the “double-line” sign, consisting of an outer sclerotic hypointense rim (red arrow) and an inner hyperintense granulation tissue (yellow arrow), which is a pathognomonic MRI pattern seen at the periphery of an area of osteonecrosis, representing the boundary between viable and non-viable bone. Sagittal fat-saturated proton-density image of the right knee redemonstrates the geographic area in the bone marrow of the medial condyle of the distal femur with abnormal increased signal intensity.

Imaging was first performed on the right knee upon presentation which revealed several small bone marrow infarcts in both femoral condyles, with predominant sclerosis and mild surrounding bone marrow edema, suggestive of AVN (Fig. 1). Imaging of the left knee followed which was also notable for distal femoral condyles’ infarcts, possible proximal tibial ischemia /infarction and no evidence of significant inflammatory joint disease (Fig. 1).

Given the inadequate response to rituximab, a decision was made to resume cyclophosphamide treatment, and prednisone (1 mg/kg) was added, later tapered gradually. Afterwards, the patient received two courses of cyclophosphamide 1 month apart, but treatment was complicated by hematuria secondary to hemorrhagic cystitis. The patient presented to the emergency room with gross hematuria. Conservative management was done with increasing hydration and the gross hematuria subsided. Clearance was then given to continue cyclophosphamide. The patient reported clinical improvement following cyclophosphamide initiation in which his toe discoloration resolved, he was able to ambulate with no noticeable pain in his knees or toes and his previous ulcers have healed and were no longer crusted.

After receiving six doses of cyclophosphamide, the patient developed nasal ulcers and was subsequently switched to infliximab (remicade) 370 mg IV at 1, 2, 6 weeks then once every 6 weeks.

He received three infliximab doses and presented for follow-up, reporting mild joint pain over both knees, increased bluish discoloration and pain over the left second toe, and worsening nasal swelling.

He was noted to have intermittent blue toes as a sequela of livedo reticularis rash, which was associated with his previous diagnosis of avascular necrosis (AVN) of both knees. Following his tenth dose of infliximab, he presented with toe cellulitis. After the eleventh dose, he complained of ankle and knee swelling, had recently experienced superficial phlebitis, denied any genital ulcers, and had a normal CRP. After this visit, infliximab treatment was increased from 370 mg every 6 weeks to 400 mg every 4 weeks.

Following treatment adjustment, the patient showed significant improvement. He no longer experienced toe discoloration or knee pain, and his previous symptoms of swelling and cellulitis did not recur. At follow-up, he reported only mild back pain, with overall better disease control. The patient is currently maintained on infliximab with no reported joint or toe pain.

Table 2 shows a summary of the case timeline and management course.

**Table 2:** Summary of case timeline and management course

Time period/Year	Clinical events/symptoms	Investigations/findings	Treatment and response
22 years ago (Age 14)	Recurrent oral aphthous ulcers and one genital ulcer → diagnosed with Behçet’s disease (BD)	—	Started on cyclophosphamide
6 years ago	Left toe discoloration and burning pain; oral ulcers; no genital ulcers	Imaging: osteomyelitis	Continued colchicine and azathioprine; resumed cyclophosphamide → improved
Few months later	Skin and soft tissue infection	—	Treated with doxycycline → resolved; cyclophosphamide resumed
2 months before presentation	Toe pain recurred	—	Switched to rituximab (1 g × 2 doses, 2 weeks apart)
Presentation	Fatigue, bilateral knee pain (no swelling or trauma)	MRI: AVN of both knees; labs: ESR 10 mm/h, CRP 17.6 mg/L; ANA/ANCA/anti-CCP negative	Switched back to cyclophosphamide + prednisone (1 mg/kg, tapered) → improved
After 2 doses	Gross hematuria	—	Diagnosed hemorrhagic cystitis → managed conservatively; continued cyclophosphamide
After 6 doses	Nasal ulcers	—	Switched to infliximab (370 mg IV at 1, 2, 6 weeks, then every 6 weeks)
Follow-up	Mild joint pain, toe discoloration, nasal swelling	—	Increased infliximab to 400 mg every 4 weeks
Recent follow-up	Only mild back pain	—	Marked improvement; maintained on infliximab
Current status	Stable disease control, no joint or toe pain, healed ulcers	—	Maintained on infliximab; regular follow-up

AVN = avascular necrosis; BD = Behçet’s disease; CRP = C-reactive protein; ESR = erythrocyte sedimentation rate; ANA = antinuclear antibody; ANCA = antineutrophil cytoplasmic antibody.

## Discussion

Our patient presents a unique case of BD with AVN of the knees, while on rituximab, not previously reported in the literature. One case series mentions bilateral AVN of the knees in six out of nine BD patients who had pulse steroids treatment^[10]^. Our patient was diagnosed with BD at the age of 14 when he presented to his pediatrician with recurrent aphthous oral and genital ulcers. He was later diagnosed with BD by his rheumatologist who noted the presence of erythema nodosum thus satisfying the diagnostic criteria set by the International Study Group for Behcet’s disease^[15]^.

Several risk factors for AVN have been identified in the literature in patients with BD. The risk of AVN is higher with the total cumulative dose of corticosteroids, rather than the daily dose^[16]^. Although corticosteroids are commonly used to treat active BD, reports of osteonecrosis or bone infarction related to BD are rare. Other risk factors include alcohol use, systemic lupus erythematosus (SLE), sickle cell disease, Gaucher’s disease, disbaric conditions (decompression sickness, “the bends”), trauma, and malignancies^[15,17]^.

Smoking might be a contributing factor to the development of AVN^[18]^. Although no studies compare the frequency of AVN between smokers and non-smokers with BD, smoking may increase the risk of AVN. Our patient smokes hubble-bubble socially and had been doing so for years when he presented with AVN.

Another factor could be the type of organ involvement in BD, specifically vascular involvement. One case in the literature reported a BD patient with unilateral knee AVN without previous corticosteroid use^[13]^. This suggests that other mechanisms, besides corticosteroids, might cause AVN in BD.

In different diseases, the pattern of joint involvement in AVN varies. In non-SLE diseases, AVN usually affects the femoral head. In SLE, it typically affects the knees and is often multifocal^[19,20]^. In BD patients, AVN most commonly affects the hip joints. Unifocal involvement is more common than multifocal involvement^[16]^. However, in our case, we observed AVN in both knee joints. In cases of secondary osteonecrosis, meaning osteonecrosis of a joint other than the one discovered on presentation, literature shows that 64% of knee cases and 55% of hip cases were bilateral^[17,21]^. This suggests that after diagnosing AVN in one joint, it may be useful to check other joints (such as the knees or hips), even if they are not symptomatic.

Diagnosis is aided by knee radiographs which are the first-line imaging studies. This is followed by MRI, which is the gold standard for characterizing osteonecrosis. This is due to its high sensitivity and specificity of nearly 100%^[22]^. Our patient underwent MRI imaging of the bilateral knees to confirm the diagnosis of osteonecrosis.

NSAIDs, limited weightbearing, quadriceps strengthening, and activity modification are the mainstay of treatment in cases of osteonecrosis of the knee^[23]^. In our reported case, control of disease severity was done in addition to conservative treatment. Given the inadequate response to rituximab, a decision was made to resume cyclophosphamide treatment, and prednisone (1 mg/kg) was added, later tapered gradually. The patient reported clinical improvement following cyclophosphamide initiation and is still in remission whilst on infliximab.

While corticosteroid exposure remains a recognized risk factor for AVN, it is important to consider the potential contribution of BD itself which is associated with vascular inflammation and a prothrombotic tendency. This may predispose patients to ischemic bone injury independently of pharmacologic therapy. Although AVN developed few months rituximab initiation in our patient, a causal role of this agent is unlikely given the delayed onset though it cannot be completely excluded. Given the complexity of overlapping risk factors, this single case cannot establish causality, and the co-occurrence of BD and AVN may be coincidental.

This report describes a single case, which limits the generalizability of the observed association between BD, its treatment and AVN. In addition, the diagnosis of AVN was based on imaging without histopathologic confirmation. Nevertheless, the case highlights the need to consider AVN as a potential complication in patients with longstanding or refractory BD.

## Data Availability

Data supporting the findings of this case report are available from the corresponding author upon reasonable request, subject to ethical and confidentiality considerations.

## Peer and provenance statement

Not commissioned; not peer reviewed.

## References

[1] Adil A Goyal A, and Quint JM. Behcet Disease, in StatPearls [Internet]. Treasure Island (FL);2025.

[2] Behcet H. Über Rezidivierende, Aphthöse, Durch Ein Virus Verursachte Geschwüre Am Mund, Am Auge Und an den Genitalien. Dermatologische Wochenschrift; 1937.

[3] Yazici H Tüzün Y Pazarli H. Influence of age of onset and patient’s sex on the prevalence and severity of manifestations of behcet’s syndrome. Ann Rheum Dis 1984;43:783–89. 6524980 10.1136/ard.43.6.783 PMC1001536

[4] de Menthon M LaValley MP Maldini C. HLA–B51/B5 and the risk of Behçet’s disease: a systematic review and meta-analysis of case–control genetic association studies. Arthritis Rheum 2009;61:1287–96. 19790126 10.1002/art.24642 PMC3867978

[5] Fazaa A Makhlouf Y Ben Massoud F. Behçet disease: epidemiology, classification criteria and treatment modalities. Expert Rev Clin Immunol 2024;20:1437–48. 39101633 10.1080/1744666X.2024.2388693

[6] Mizushima Y. Behcet’s disease. Curr. Opin. Rheumatol 1991;3:32–35 2043450

[7] Lavalle S Caruso S Foti R. Behcet’s disease, pathogenesis, clinical features, and treatment approaches: a comprehensive review. Medicina (Kaunas) 2024;60:562 38674208 10.3390/medicina60040562 PMC11051811

[8] Belfeki N Ghriss N Fourati M. Neuro-Behcet’s disease: a review. Rev Med Interne 2024;45:624–33. 38937151 10.1016/j.revmed.2024.06.007

[9] Emmi G Bettiol A Hatemi G. Behcet’s syndrome. Lancet 2024;403:1093–108. 38402885 10.1016/S0140-6736(23)02629-6

[10] Atas N Bitik B Varan O. Clinical characteristics of avascular necrosis in patients with Behcet disease: a case series and literature review. Rheumatol Int 2019;39:153–59. 30560445 10.1007/s00296-018-4224-9

[11] Bosco G Vezzani G Enten G. Femoral condylar necrosis: treatment with hyperbaric oxygen therapy. Arthroplast Today 2018;4:510–15. 30560184 10.1016/j.artd.2018.02.010 PMC6287235

[12] Yapar Z Kbar M Soy MK. Osteonecrosis in behcet’s disease seen on bone scintigraphy. Clin Nucl Med 2001;26:267–68. 11245135 10.1097/00003072-200103000-00029

[13] Chang HK Choi YJ Baek SK. Osteonecrosis and bone infarction in association with Behcet’s disease: report of two cases. Clin Exp Rheumatol 2001;19:S51–4. 11760400

[14] Ahmed K Al-Jabir A Mathew G. Revised surgical CAse REport (SCARE) guideline: an update for the age of Artificial Intelligence. Prem J Sci 2025;10:100079.

[15] Criteria for Diagnosis of Behçet’s Disease. International Study Group for Behçet’s Disease. Lancet 1990;335:1078–80. 1970380

[16] Nejadhosseinian M Babagoli M Faezi ST. Osteonecrosis as a rare musculoskeletal complication in Behcet’s disease- the largest case series with literature review. BMC Rheumatol 2023;7:42 38031147 10.1186/s41927-023-00366-3 PMC10687826

[17] Boontanapibul K Steere JT Amanatullah DF. Initial presentation and progression of secondary osteonecrosis of the knee. J. Arthroplasty 2020;35:2798–806. 32527695 10.1016/j.arth.2020.05.020

[18] Matsuo K Hirohata T Sugioka Y. Influence of alcohol intake, cigarette smoking, and occupational status on idiopathic osteonecrosis of the femoral head. Clin Orthop Relat Res 1988;234:115–23. 3409564

[19] Mont MA Glueck CJ Pacheco IH. Risk factors for osteonecrosis in systemic lupus erythematosus. J Rheumatol 1997;24:654–62. 9101497

[20] Oinuma K Harada Y Nawata Y. Osteonecrosis in patients with systemic lupus erythematosus develops very early after starting high dose corticosteroid treatment. Ann. Rheum. Dis 2001;60:1145–48. 11709458 10.1136/ard.60.12.1145 PMC1753447

[21] Boontanapibul K Steere JT Amanatullah DF. Diagnosis of osteonecrosis of the femoral head: too little, too late, and independent of etiology. J. Arthroplasty 2020;35:2342–49. 32456965 10.1016/j.arth.2020.04.092

[22] Expert Panel on Musculoskeletal Imaging, Ha AS Bartolotta CE Bucknor RJ. ACR appropriateness criteria® osteonecrosis: 2022 update. J Am Coll Radiol 2022;19: 409–16 10.1016/j.jacr.2022.09.009 36436966

[23] Zmerly H Moscato M Akkawi I. Treatment options for secondary osteonecrosis of the knee. Orthop Rev (Pavia) 2022;14:33639 35775038 10.52965/001c.33639 PMC9239350

